# Acceptance of a mental health app (JoyPop^TM^) for postsecondary students: a prospective evaluation using the UTAUT2

**DOI:** 10.3389/fdgth.2025.1503428

**Published:** 2025-02-18

**Authors:** Ishaq Malik, Aislin R. Mushquash

**Affiliations:** Department of Psychology, Lakehead University, Thunder Bay, ON, Canada

**Keywords:** mental health app, postsecondary students, acceptance, UTAUT2, JoyPop^TM^, mobile health, digital health, emotion regulation

## Abstract

**Introduction:**

Mental health (MH) smartphone applications (MH apps) can support the increasing MH needs of postsecondary students and mitigate barriers to accessing support. Evaluating MH app acceptance using technology acceptance models is recommended to improve student engagement with MH apps. The JoyPop^TM^ app was designed to improve youth resilience and emotion regulation. The JoyPop^TM^ app is associated with improved student MH, but its acceptance has yet to be evaluated quantitatively. The present study used the Unified Theory of Acceptance and Use of Technology (UTAUT2) to evaluate and examine constructs and moderators influencing the acceptance (i.e., behavioural intention) and use of the JoyPop^TM^ app.

**Method:**

Participants were 183 postsecondary students attending a Canadian University who used the app for one week and completed measures before and after using the app. Relationships posited by the UTAUT2 were tested using partial least squares structural equation modelling (PLS-SEM).

**Results:**

Most participants accepted the JoyPop^TM^ app. The UTAUT2 model explained substantial variance in behavioural intention and app use. Performance expectancy, hedonic motivation, and facilitating conditions predicted behavioural intention, and behavioural intention and facilitating conditions predicted app use. Age moderated the association between facilitating conditions and behavioural intention. Experience moderated the relationship between performance expectancy, hedonic motivation, and social influence on behavioural intention.

**Discussion:**

Results provide insight into factors influencing the acceptance of the JoyPop^TM^ app and its ability to engage students. Results also provide valuable insights for evaluating and optimally designing MH apps.

## Introduction

Despite the benefits and unique opportunities postsecondary education provides (1–3), mental health (MH) difficulties among Canadian students are high and increasing (4–6). A national survey examining the MH of 11,322 students across 32 Canadian colleges and universities found that many reported moderate to serious levels of psychological distress (85.4%), high levels of loneliness (58.6%), suicidal ideation (35.5%), and a diagnosis of an anxiety (32%) or mood (24.6%) disorder (4). Unaddressed MH difficulties can increase student distress and risk behaviours, negatively impact academic performance and well-being, and have long-lasting personal, social, and economic consequences (5, 7–9). Numerous stressors and challenges contribute to student MH difficulties, such as academic demands, concerns about the future, finances, and relationships (4, 5, 10). Students' abilities to cope with these stressors and the support they receive significantly impact MH and life outcome trajectories (5, 9, 11). In response to rising student MH needs, various discrete interventions (e.g., mindfulness interventions) and local, provincial, and national MH initiatives have been developed and implemented across Canadian postsecondary institutions (12, 13). However, students continue to face numerous structural and attitudinal barriers to accessing support, which reduces help-seeking and the adequacy of current MH services to meet student needs. Commonly reported barriers include minimal long-term therapy and off-site support, long wait times, lack of availability, time and financial constraints, and stigma (14–16). Consequently, there has been a growing interest in Canada in developing novel prevention and intervention efforts that expand the reach, scalability, affordability, and flexibility of MH supports (17).

Mobile Health (mHealth) smartphone applications designed to support MH (MH apps) are touted as a practical method to expand student MH support and mitigate barriers to accessing care (17, 18). MH apps are especially promising tools for students because of students' high rates of smartphone use and openness to using apps (17, 19, 20). MH apps are discussed as a solution to mitigate barriers to care because they are more scalable than traditional MH services and can provide 24-h on-demand support (12, 18). Further, integrating MH apps into existing services can increase availability and access to support and reduce stigma, costs, and MH disparities (17, 18, 21, 22). Among postsecondary students, research shows that MH apps are effective in improving outcomes, such as depression, anxiety, stress, well-being, and academic performance (23, 24). However, student engagement and long-term uptake of MH apps are low (25, 26). For example, a study in a sample of 742 postsecondary students found that among participants who had previously used a MH app, only 2.4% sustained use for four weeks or more (27). Evidence of effectiveness is also not a strong predictor of engagement and use (19, 28). Increasing user engagement is essential to increase the overall impact of MH apps for student MH (26, 28), and understanding user acceptance, which strongly affects engagement, is critical to improving engagement and use of MH apps (26, 28). Using technological acceptance frameworks to examine factors influencing user acceptance is recommended because of their utility in predicting and understanding engagement and continued use of mHealth apps (24, 28, 29–32).

The most prominent technology acceptance frameworks predicting acceptance and use of mHealth apps are the Technology Acceptance Model (TAM) (33), the Unified Theory of Acceptance and Use of Technology (UTAUT) (34), and the extended Unified Theory of Acceptance and Use of Technology (UTAUT2) (35). These frameworks are all based on psychological theories (e.g., Theory of Reasoned Action) (36) and conceptualize acceptance as users' *behavioural intention to use technology*, which is argued to influence use (35, 37). The UTAUT2 has become the leading framework because it captures the consumer/individual perspective, considers organizational, individual, and social factors, and explains more variance in behavioural intention and use of technologies (35, 38, 39).

The UTAUT2 is an extension of the original UTAUT model (35). It includes seven core constructs (35). Performance expectancy refers to users' expectations that a technology is useful for its purpose. Effort expectancy relates to the ease of use and user-friendliness of technology. Social influence is the degree to which important people believe they should use technology. Facilitating conditions refers to the degree to which users believe they have sufficient organizational and technical infrastructure to use technology (34, 35). Hedonic motivation refers to the fun, pleasure, and enjoyment users experience with technology. Price value examines the influence of cost and pricing structure use. Habit refers to the automaticity with which people perform behaviours because of learning (35). All constructs in the model are purported to influence users' behavioural intention to use a technology. Behavioural intention, facilitating conditions and habit are posited to influence use directly. The UTAUT2 includes age, gender, and user experience with a specific technology as moderators of core constructs on behavioural intention, and experience is a moderator between behavioural intention and use (35). The UTAUT2 has been used and validated across diverse populations, countries, and contexts to evaluate and understand mHealth app acceptance (31, 39). UTAUT2 constructs and moderator variables significantly influence behavioural intention and use of mHealth apps in samples of postsecondary students (40–42).

Evaluating user acceptance, especially when guided by technology acceptance models, is critical in determining the relative importance of acceptance factors and informing app adaptations to increase user engagement and overall impact (24, 30, 43). However, among the many MH apps available on major marketplaces, few have been quantitatively evaluated regarding their acceptance (22, 26), and evaluations of MH app acceptance among postsecondary students are even more limited (22, 24, 26). Prior research implementing UTAUT2 constructs to quantitatively evaluate MH app acceptance is also cross-sectional, relies on retrospective reports of participants' experiences with groups of MH apps rather than gleaning users' experiences after using a specific app, and does not include all UTAUT2 constructs and moderators (40, 44, 45). Consequently, there is a significant need to quantitatively evaluate and understand the acceptance of specific MH apps among students, as the purpose and features of different MH apps are likely to vary. These evaluations can better assess the relative importance of acceptance factors to the unique characteristics of individual MH apps. These limitations informed the purpose of the present study, which is to evaluate and better understand factors influencing the acceptance of a resilience-building MH app (JoyPop^TM^) among a diverse student sample. This research will help determine whether the JoyPop^TM^ app can be a valuable and engaging tool to support student MH, and results will inform future app adaptations.

**Figure 1:**
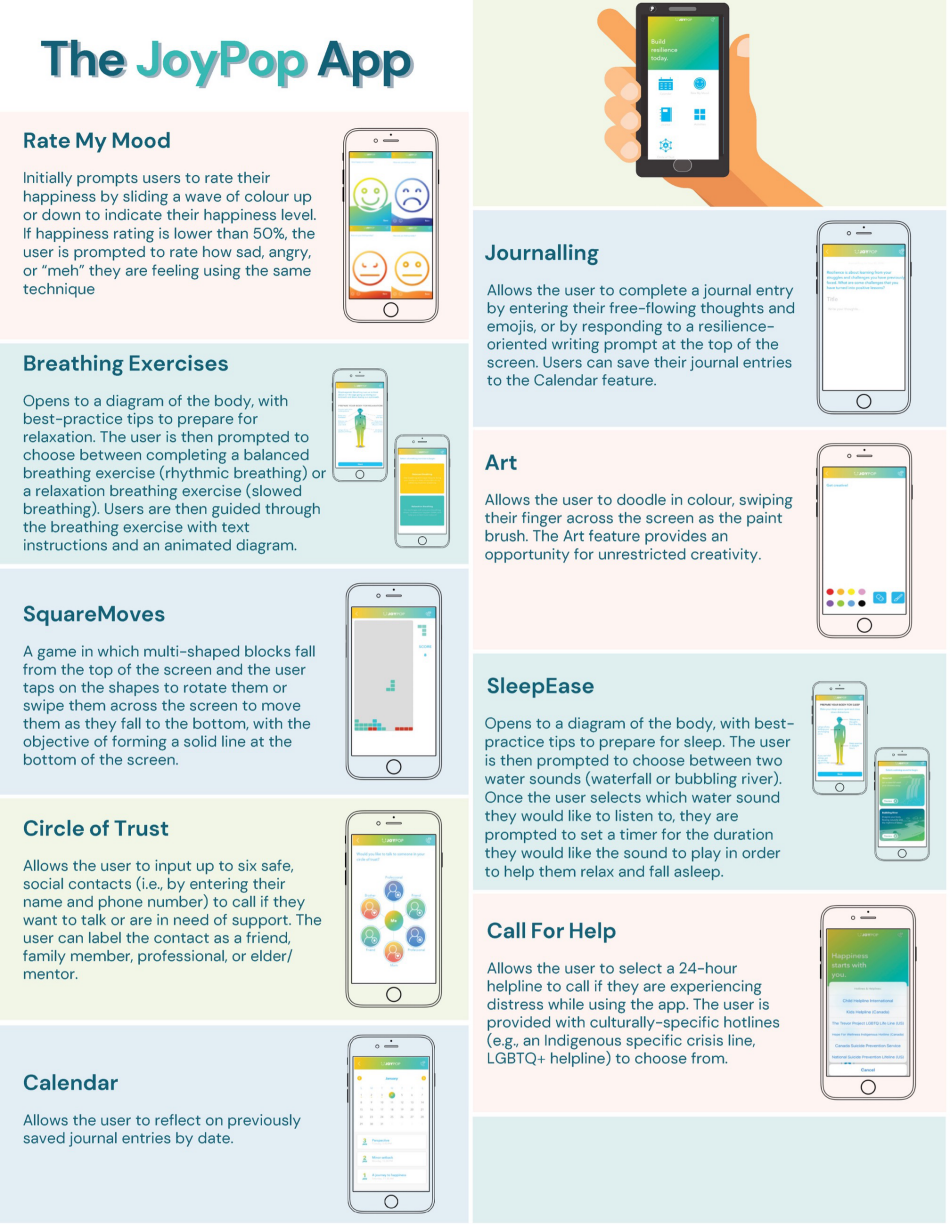
Summary and highlights of features in the JoyPop^TM^ app.

The JoyPop^TM^ app was designed for youth (12+) and emerging adults (46). It contains multiple evidence-based features (see Figure 1) to support adaptive coping and emotion regulation (46, 47). The JoyPop^TM^ app has a growing multimethod evidence base among diverse samples of youth and young adults in clinical and non-clinical settings (47–52). Research among postsecondary students suggests that using the app is associated with improved emotion regulation, stress, and depressive symptoms (47, 51). Students also perceive that the app increases self-regulation opportunities and self-awareness (52). However, the acceptance of the JoyPop^TM^ app among students has yet to be quantitatively evaluated using an established technology acceptance model.

Considering this limitation in the JoyPop**^TM^** app literature, the paucity of quantitative MH acceptance evaluations among postsecondary students, and that no studies have incorporated the UTAUT2 model to quantitatively examine the acceptance of a specific MH app despite its utility in evaluating mHealth app acceptance, our objectives were to employ the UTAUT2 model to 1) quantitatively evaluate students’ acceptance of the JoyPop^TM^ app and 2) examine factors influencing the app's acceptance and use. Because no study has examined the acceptance of a specific MH app using the UTAUT2, we based our hypotheses on the broader literature and the original UTAUT2 results (31, 35, 39). We did not include price value because the JoyPop^TM^ app was freely available at the time of the study. We hypothesized that:

H1: Performance expectancy, effort expectancy, social influence, facilitating conditions, hedonic motivation, and habit would significantly predict behavioural intention to use the JoyPop^TM^ app.
H2: Behavioural intention to use the JoyPop^TM^ app, facilitating conditions, and habit would significantly predict use of the app.
H3: Age, gender, and experience with MH apps (“experience”) would moderate the relationship between UTAUT2 constructs and behavioural intention to use the JoyPop^TM^ app.
H4: Experience would moderate the relationship between behavioural intention to use the JoyPop^TM^ app and use of the app.

## Method

### Procedure

This study was reviewed and approved by the Research Ethics Board at Lakehead University. Students were eligible for the study if they were enrolled at Lakehead University and fluent in English. We selected this sample because students have increasing MH needs and face many barriers to accessing support, which makes them a relevant group to evaluate the utility of MH apps. We recruited students throughout the fall (2023) and winter (2024) academic semesters. Several measures were taken to increase the representativeness of our sample and minimize sampling bias. First, we used various recruitment methods to reach a wide range of students by posting flyers around campus, announcing our study to classes, providing postcards to students after class, emailing students in classes (e.g., through course instructors), and posting our study online via the Department of Psychology's undergraduate psychology participant pool site. Second, to improve the diversity of our sample, we had no restrictions on participant characteristics, such as year of study or program. Finally, to minimize biases of excluding participants without an iOS device, we participants without a suitable device with a refurbished iPhone containing only the app.

We used a one-week prospective design to ensure participants had sufficient time to use the app and provide informed evaluations regarding their acceptance. This study had three parts. For Part 1, participants attended an orientation session where they provided informed consent, completed demographic and MH app experience measures, and received the app. In Part 2, we reminded participants (via text/email) twice a day to use the app for one week. In Part 3, participants completed online measures assessing UTAUT2 constructs and app use. We compensated participants via cash/e-transfer or bonus course marks towards an eligible psychology course for completing measures ($10 for Part 1 or one bonus point; $20 for Part 3 or 1.5 bonus points).

#### Sample size estimation

In PLS-SEM, the ten times rule (i.e., sample size based on ten times the number of independent variables in the structural model) is often used to estimate the minimum required sample size. However, this approach underestimates the sample size needed for sufficient power and does not consider the entire model being assessed (53, 54). Thus, we followed the most recent recommendations and used the inverse square root method to determine the minimum sample size needed because it provides the most conservative and accurate estimate for PLS-SEM models (53, 54). This method bases sample size on the smallest path coefficient one expects to be significant, determined from research with similar conceptual models (53, 54). Past research employing the UTAUT2 to evaluate the acceptance of various types of mHealth apps (which included MH apps) found that the smallest significant path coefficient at the *p* < 0.05 level was 0.19 in the relationship between social influence and behavioural intention (40). Using minimum sample size requirement tables derived from the inverse square root method found in Hair et al. (53), to detect a minimum significant path coefficient between 0.11 and 0.20, we required a minimum sample size of 155 for 80% power at the 5% significance level. Considering ∼12% attrition rates in a similar JoyPop^TM^ study conducted by MacIsaac et al. (47), we aimed to recruit at least 175 participants to account for estimated attrition throughout the study.

### Participants

Our final sample consisted of 183 participants (see Table 1). Most participants were women (82%), with a mean age of 22.60 (*SD* = 7.08, 71% between 16 and 21, range = 16–56). Most participants identified as White (54.6%), followed by Black (18%) and South Asian (10.9%). Most participants were completing their first (42.6%) or second year (29.5%) in nursing (36.1%) or psychology programs (33.1%). Most participants used the app every day of the study period (*M* = 4.69, *Mdn* = 5, *SD* = 0.81).

**Table 1:** Participant demographics.

	*n* (%)
Gender	
Women	150 (82)
Men	33 (18)
Ethnicity	
White	100 (54.6)
Black	33 (18)
South Asian	20 (10.9)
Indigenous	10 (5.5)
Southeast Asian	7 (3.8)
Other (e.g., Middle Eastern, South American, Asian Canadian)	13 (7.2)
Country of Birth	
Canada	116 (63.3)
Nigeria	26 (14.2)
India	14 (7.7)
Pakistan	4 (2.2)
Philippines	3 (1.6)
Other (e.g., Italy, Barbados, Vietnam, Sri Lanka, Uganda, China)	20 (11.1)
Program	
Nursing	66 (36.1)
Psychology	62 (33.9)
Education	10 (5.5)
Social Work	7 (3.8)
Computer Science	7 (3.8)
Biology	7 (3.8)
Other (e.g., Kinesiology, Business, Outdoor Rec, Political Science)	24 (13.1)
Year of university	
1	81 (44.2)
2	54 (29.5)
3	26 (14.2)
4	19 (10.4)
>5	3 (1.6)

Most participants had not used app-based MH care (80.9%). Of those who had used app-based MH care (19.1%), most used it for 0–6 months (62.9%), followed by not recalling the amount of time (20%) and 1–2 years (14.3%). Most participants had no MH apps on their phones/devices (86.3%). Among participants who reported having a MH app on their phone/device (13.7%), 88% had 1–3 apps, and 8% had more than seven apps. Frequency of use among these participants showed that 60% reported rarely using these apps, 12% reported using them once a week, 12% reported 2–3 times a week, and 16% used them once or twice a day.

### Measures

#### UTAUT2 constructs

We used the UTAUT2 scale developed by Venkatesh et al. (35) to measure seven core constructs of the UTAUT2 model and behavioural intention to use the JoyPop^TM^ app with 23 items slightly adapted to fit the context of the present study (see Supplementary File S1): Performance Expectancy (three items), Effort Expectancy (four items), Social Influence (three items), Facilitating Conditions (four items), Hedonic Motivation (three items), Habit (three items), and Behavioural Intention (three items). We adapted items where appropriate (i.e., changing “mobile internet” to “the JoyPop^TM^ app”). We adapted one item from the Performance Expectancy construct to better fit the intended use of the JoyPop^TM^ app (i.e., we changed “Using mobile internet increases my productivity,” to “Using the JoyPop^TM^ app improves my mental health and/or productivity”). We adapted one item from the Habit construct based on a previous adaptation by Wu et al. (55) (i.e., we changed “I am addicted to using mobile internet” to “I am immersed in using/accepting the JoyPop^TM^ app”). Participants rated items on a seven-point Likert scale ranging from 1 (strongly disagree) to 7 (strongly agree). We calculated scores for each construct by averaging corresponding items. We used participants' scores on the behavioural intention construct scale to evaluate the overall acceptance of the JoyPop^TM^ app (32, 35).

Both the original and adapted versions of the UTAUT2 scale show strong psychometric properties in postsecondary student samples (35, 39, 40, 42). The internal consistency of construct scales in the present study were adequate (performance expectancy, *α* = .90; effort expectancy, *α* = .74; social influence, *α* = .91; facilitating conditions, *α* = .57; hedonic motivation, *α* = .91; habit, *α* = .81; behavioural intention, *α* = .94).

#### App use

To examine app use, we used an item (“How many times do you think you would use the JoyPop^TM^ app in the next 12 months if it was relevant to you”) from the Subjective Quality scale of the User Version of the Mobile Application Rating Scale (uMARS) (56). Participants responded on a five-point scale ranging from 1 (None) to 5 (>50 times). We used this item because it is conceptually similar to the original *use* question on the UTAUT2 questionnaire and was developed to assess the frequency of mHealth app use.^1^

### Data analysis

We coded whether participants had used app- or computer-based MH care as a binary categorical variable to examine the moderating effect of experience. We reported frequencies of participant scores on the behavioural intention construct scale to facilitate the interpretation of the app's overall acceptance.

#### PLS-SEM

We used the SmartPLS version 4 software (57) to conduct Partial Least Square Structural Equation Modelling (PLS-SEM) and examine hypothesized predictive-causal relationships (H1 to H4). We chose PLS-SEM because it is the preferred method of analysis when a study aims to test the predictive capability of a complex theoretical causal-predictive model that includes moderating effects and many constructs (six or more) and relationships (53, 58, 59). The causal-predictive nature makes PLS-SEM especially useful when deriving recommendations (53, 58), such as informing future adaptions to the JoyPop^TM^ app to improve acceptance.

We evaluated two key components within the PLS-SEM model: a measurement model (outer model) and a structural model (inner model) (53). Throughout our analysis, we based our evaluation criteria and decision-making processes on the most recent PLS-SEM guidelines, recommendations, and research summarized by Hair et al. (53) and Sarstedt et al. (60).

##### Measurement model evaluation

We assessed the reliability of construct indicators (i.e., items used to measure constructs) and the internal consistency reliability, convergent validity, and discriminant validity of each construct in the model. We removed indicators and constructs that did not meet reliability and validity evaluation criteria to ensure that quality measures were used in the structural model (53). We evaluated indicator reliability by examining the outer loadings of constructs. Outer loadings should be >0.70 (53, 60). We removed indicators with outer loadings below 0.40 (53, 60). We considered indicators with outer loadings between ≥0.40 and <0.70 for removal if removing them increased construct reliability or validity to recommended thresholds and did not compromise content validity. We retained indicators with outer loadings within these ranges of values if the indicator's construct met the recommended thresholds for internal consistency reliability, convergent validity, and discriminant validity (53, 60).

We assessed internal consistency reliability via Cronbach's *α*, composite reliability (*ρ*_C_), and an exact reliability coefficient (*ρ*_A_), which estimates the reliability of construct scores using the construct's weights rather than loadings (53, 60, 61). All internal consistency estimates should be ≥0.70 (53, 60). We assessed convergent validity by computing the average variance extracted (AVE) for all items for each construct. AVE's ≥ 0.50 are recommended (53, 60). We examined the discriminant validity of constructs via the heterotrait-monotrait ratio (HTMT) because it is more reliable and accurate than other common methods, such as the Fornell-Larcker criterion (53, 60). We used the <0.90 cut-off value, which is recommended when constructs are conceptually similar (60, 62).

##### Structural model evaluation

We used the structural model to test our hypotheses that the UTAUT2 constructs would predict behavioural intention (H1), and facilitating conditions and behavioural intention would predict use of the JoyPop^TM^ app (H2). We first checked for collinearity by examining the variance inflation factor (VIF). VIFs are recommended to be ≤3 because this indicates no collinearity issues (53, 60). We used the adjusted coefficient of determination (*R*^2^_adj_) to examine our structural model's explanatory power (53, 60). *R*^2^ values of 0.75, 0.50 and 0.25 are considered substantial, moderate, or weak (53, 58). We conducted a one-tailed test at the 5% significance level with PLS-SEM's nonparametric bootstrap procedure (10,000 iterations) to estimate path coefficients and their associated *t* values, *p* values, and confidence intervals (53, 63). We employed the Bias-Corrected and accelerated (BCa) bootstrap method to account for potential bias arising from asymmetrical bootstrap distributions for parameter estimates (60, 64). We examined the unique impact of predictors using *f^2^* effect sizes. We used Cohen's (65) guidelines for interpreting the *f^2^*effect size, with values of 0.02, 0.15, and 0.25 representing small, medium, and large effects.

Because *R^2^* values are derived from all data used for model estimation and do not provide information about the model's prediction power outside of the sample data included in the initial calculation of the model, they are not considered true estimates of predictive power (a model's utility in predicting new observations) (53, 59, 64). Consequently, if our hypothesized structural model was significant, we planned to test its out-of-sample prediction power using the novel and recommended PLS_predict_ procedure option in SmartPLS 4 (53, 66). The PLS_predict_ procedure first estimates the predictive power of a structural model by generating model estimates from a subset of observations (training sample). These estimates are applied to a subset of values on predictor variable indicators that have been omitted when generating parameter estimates (holdout sample), which are then used to predict dependent construct indicators within the holdout sample (64, 66). The strength of a model's out-of-sample prediction power is assessed by comparing prediction errors [the root mean squared errors (RMSEs)] produced by the PLS-SEM model to those made by a naïve linear regression model (LM) (64, 67). This method determines whether a theoretical model improves (or, at minimum, does not worsen) the predictive utility of available data among indicators (53, 67). See Supplementary Table S1 for guidelines for interpreting PLSpredict results [also see Shmueli et al. (67), for a thorough description of the PLS_predict_ method].

##### Moderation analysis

To test our hypothesis that age would moderate the relationships between the UTAUT2 constructs and behavioural intention (part of H3), we created interaction terms between age and relevant predictor variables using the recommended two-stage approach (53, 60, 68). Because gender and experience were grouping variables, our method to test the hypotheses that gender and experience would moderate the associations between UTAUT2 constructs and behavioural intention (part of H3), and experience would moderate the influence of behavioural intention on app use (H4) depended on whether there was full or partial measurement variance across groups (69). We employed the Measurement Invariance of Composite Models (MICOM) procedure in SmartPLS 4 to assess partial or full measurement invariance [see Henseler et al., (69) and Cheah et al., (70)]. If partial measurement invariance was confirmed, we planned to use the recommended multigroup analysis (MGA) procedure, to test whether there were significant differences between group-specific parameter estimates (53, 70). If full measurement invariance was confirmed, we followed suggestions to pool data from groups and account for structural heterogeneity by creating interaction terms between moderating variables and relevant latent constructs using the two-stage approach (53, 69, 70).

To determine the strength of the moderating effects, we calculated and interpreted the *f^2^* effect sizes (53, 60). We interpreted *f^2^* values as 0.005 (small), 0.01 (medium), and 0.025 (large) because standard cutoff values delineating *f^2^*effect sizes are less relevant when interpreting interaction terms (53, 71). We created simple slope plots to facilitate the interpretation of significant moderating effects (53, 72).

## Results

### Descriptives and overall acceptance

We present descriptive statistics and correlations for UTAUT2 constructs and app use in Table 2. Most participants showed moderate to strong acceptance of the JoyPop^TM^ app. Specifically, 45.4% of participants indicated moderate (somewhat agree) to strong (strongly agree) acceptance of the app (range: 4.67–7), 25.1% showed low levels of acceptance (strongly disagree to somewhat disagree; range: 1–3.33), and 23% were unsure whether they intended to use the app (range: 3.67–4.33).

**Table 2:** Descriptive statistics and correlations for UTAUT2 constructs and App Use.

	*M*	SD	PE	EF	FC	SI	HT	HM	BI	App use
PE	4.47	1.39	1.00							
EF	6.25	0.72	.39	1.00						
FC	5.67	0.94	.29	.41	1.00					
SI	4.01	1.21	.64	.24	.24	1.00				
HT	4.29	1.39	.82	.43	.33	.61	1.00			
HM	5.27	1.31	.75	.51	.33	.47	.78	1.00		
BI	4.46	1.57	.82	.37	.43	.52	.85	.76	1.00	
App Use	3.55	1.61	.62	.33	.18	.50	.60	.59	.67	1.00

PE, performance expectancy; EF, effort expectancy; FC, facilitating conditions; SI, social influence; HT, habit; HM, hedonic motivation; BI, behavioural intention.

### PLS-SEM

#### Measurement model

**Figure 2:**
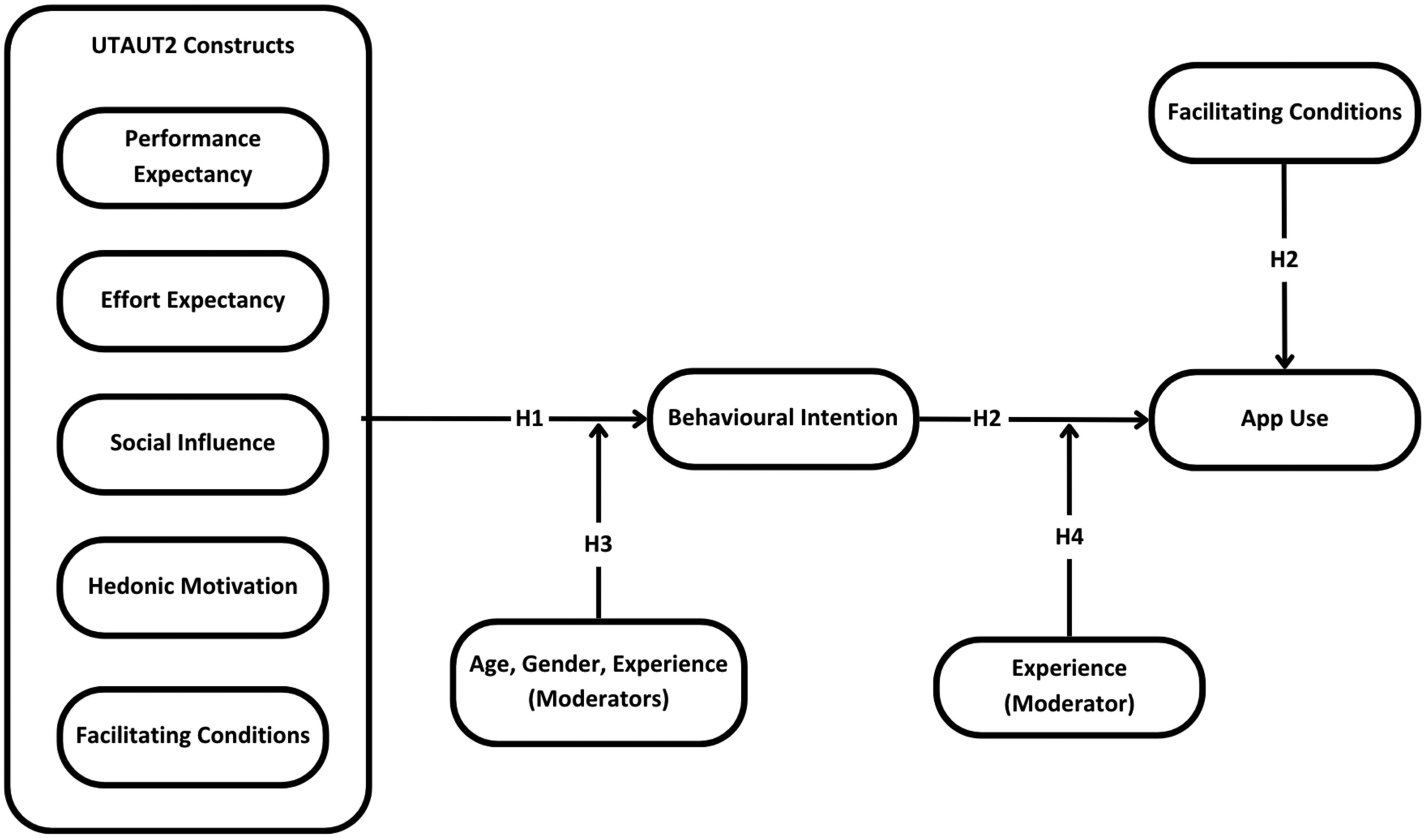
Final structural model and tested hypotheses.

We removed two indicators on the facilitating conditions construct with outer loadings below 0.70 (0.39 and 0.66), which improved the reliability and convergent validity to recommended thresholds. We retained one indicator on the effort expectancy construct with an outer loading of 0.64 because the construct met all evaluation criteria for reliability and convergent validity. Discriminant validity was not established for the habit construct (HTMTs > 0.90), and we removed it from the model. Thus, we did not examine the hypothesized relationships between habit and behavioural intention (part of H1) and habit and use (part of H2) in the structural model. Our final model (see Figure 2) included performance expectancy, effort expectancy, facilitating conditions, social influence, hedonic motivation, behavioural intention, and app use because all constructs met the measurement model reliability and validity evaluation criteria. We present the outer loadings of indicators, the internal consistency reliability and convergent validity estimates of constructs in Table 3 and the discriminant validity of constructs in Table 4.

**Table 3:** Final model outer loadings, internal consistency, and convergent validity.

Construct and indicators	Outer loadings	*α*	*ρ_A_*	*ρ_C_*	AVE
Behavioural intention (BI)		0.94	0.94	0.96	0.90
BI1	0.94				
BI2	0.94				
BI3	0.96				
Performance expectancy (PE)		0.90	0.90	0.94	0.84
PE1	0.92				
PE2	0.91				
PE3	0.92				
Effort expectancy (EF)		0.77	0.86	0.85	0.58
EF1	0.64				
EF2	0.84				
EF3	0.84				
EF4	0.70				
Facilitating conditions (FC)		0.70	0.86	0.86	0.76
FC1	0.80				
FC3	0.94				
Social influence (SI)		0.91	0.91	0.94	0.85
SI1	0.89				
SI2	0.93				
SI3	0.94				
Hedonic motivation (HM)		0.92	0.92	0.95	0.86
HM1	0.93				
HM2	0.94				
HM3	0.91				

Cronbach's Alpha (*α*); Reliability Coefficient (*ρ_A_*); Composite Reliability (*ρ_C_*); Average Variance Extracted (AVE).

**Table 4:** Heterotrait-Monotrait-Ratios (HTMTs) of constructs in the final model.

	BI	EF	FC	HM	PE	SI
BI						
EF	0.41					
FC	0.43	0.33				
HM	0.82	0.60	0.28			
PE	0.89	0.46	0.24	0.82		
SI	0.57	0.28	0.22	0.52	0.71	
App use	0.69	0.35	0.11	0.61	0.66	0.52

PE, performance expectancy; EF, effort expectancy; FC, facilitating conditions; SI, social influence; HT, habit; HM, hedonic motivation; BI, behavioural intention.

#### Structural model

Once the measurement quality of the model was established, we evaluated and interpreted the structural model to test our hypotheses that each UTAUT2 construct would predict behavioural intention (H1), and facilitating conditions and behavioural intention would predict app use (H2). We found no collinearity issues (all VIFs ≤ 3; see Table 5). Overall, our model explained a significant and substantial amount of variance in behavioural intention [*R^2^*_adjusted_ = 0.751, *p* < .001, 95% CI (0.679, 0.795)]. Performance expectancy (*β* = 0.576, *p* < .001, *f^2^ =* 0.457; large effect), hedonic motivation (*β* = 0.334, *p* < .001, *f^2^ =* 0.173; medium effect), and facilitating conditions (*β* = 0.181, *p* < .001, *f^2^ =* 0.124; small effect) significantly and positively predicted behavioural intention. Social influence and effort expectancy did not significantly predict behavioural intention. Therefore, we found partial support for H1.

**Table 5:** Variance inflation factor (VIF) statistics for the structural model.

Model paths	VIF
Effort expectancy -> behavioural intention	1.42
Facilitating conditions -> behavioural intention	1.10
Hedonic motivation -> behavioural intention	2.67
Performance expectancy -> behavioural intention	3.00
Social influence -> behavioural intention	1.71
Facilitating conditions -> app use	1.16
Behavioural intention -> app use	1.16

Behavioural intention and facilitating conditions explained a moderate amount of variance in use [*R^2^*_adjusted_ = 0.460, *p* < .001, 95% CI (0.367, 0.541)]. Behavioural intention (*β* = 0.725, *p* < 0.001, *f^2^ =* 0.849; large effect) and facilitating conditions (*β* = 0.131 *p* < .001, *f^2^ =* 0.038; small effect) significantly and positively predicted use. Thus, H2 was partially supported because we did not examine the influence of habit on use (see Table 6). Considering the significance of the structural model, we assessed the model's predictive power. Our model showed high predictive power because, compared to the naïve LM benchmark, lower RMSEs were produced by the PLS-SEM model on all indicators (see Table 7).

**Table 6:** Structural model path estimate results.

Hypothesis #	Relationship	Path Coefficient (*β)*	*t-*values	CI	Decision	Overall Hypothesis
H1	PE -> BI	0.576	8.265	[0.453, 0.683]**	Supported	Partially supported
	HM -> BI	0.334	4.795	[0.225, 0.455]**	Supported	
	FC -> BI	0.181	3.801	[0.103, 0.259]**	Supported	
	EF -> BI	−0.065	1.526	[−0.135, 0.004]	Not supported	
	SI -> BI	−0.026	0.478	[−0.119, 0.057]	Not supported	
	HT -> BI				Untested	
H2	BI -> app use	0.725	16.934	[0.650, 0.792]**	Supported	Partially supported
	FC -> app use	0.131	3.649	[0.075, 0.193]**	Supported	
	HT -> app use				Untested	

PE, performance expectancy; EF, effort expectancy; FC, facilitating conditions; SI, social influence; HT, habit; HM, hedonic motivation; BI, behavioural intention; CI, 95% Bootstrap Confidence Intervals.

** *p* < 0.001.

**Table 7:** PLS_predict_ results.

Indicators of the outcome variable	PLS model RMSE	Naïve (LM) benchmark model RMSE
BI1	**0**.**958**	1.011
BI2	**0**.**974**	1.011
BI3	**0**.**982**	1.020
App use	**0**.**907**	0.916

Bolded Root Mean Squared Errors (RMSEs) indicate better predictive power for the PLS-SEM model among an indicator of the outcome variable compared to the Naïve Linear Regression Model (LM) Benchmark; Behavioural Intention (BI).

#### Moderation analysis

We first examined interaction effects for age to test our hypothesis that it would moderate associations between UTAUT2 constructs and behavioural intention. Age only showed a significant and large positive moderating effect on the relationship between facilitating conditions and behavioural intention (*f^2^ =* 0.032). We found full measurement invariance for experience and included it as an interaction effect to test our hypotheses that experience would moderate the relationship between UTAUT2 constructs and behavioural intention, as well as behavioural intention and use. Experience showed a significant and large positive effect on the relationship between performance expectancy and behavioural intention (*f^2^ =* 0.088) and significant and large negative effects on the relationships between hedonic motivation and behavioural intention (*f^2^ =* 0.059) and social influence and behavioural intention (*f^2^ =* 0.040; see Table 8). We found partial measurement invariance for gender and conducted a MGA to test our hypothesis that gender would moderate the relationship between UTAUT2 constructs and behavioural intention. We found no significant differences in path coefficients between women and men (see Table 9). In sum, H3 was partially supported because age, experience, and gender did not moderate all proposed hypothesized relationships between UTAUT2 constructs and behavioural intention. H4 was not supported because experience did not moderate the relationship between behavioural intention and use.

**Table 8:** Moderating effects of age and mental health app experience.

Interaction term relationships	Path coefficients	*t*-values	Lower 95% (CI)	Upper 95% (CI)	*p*-value
Age × EF -> BI	−0.080	0.891	−0.268	0.040	0.186
Age × FC -> BI	0.092	1.679	0.021	0.209	**0**.**047**
Age × FC -> Use	−0.032	0.515	−0.133	0.075	0.303
Age × HM -> BI	0.083	0.627	−0.084	0.385	0.265
Age × PE -> BI	−0.131	1.251	−0.369	0.001	0.105
Age × SI -> BI	0.095	1.167	−0.045	0.216	0.122
Experience × BI -> app use	0.085	0.496	−0.215	0.350	0.310
Experience × EF -> BI	−0.166	0.749	−0.521	0.185	0.227
Experience × FC -> BI	−0.151	1.408	−0.321	0.025	0.080
Experience × FC -> app use	0.028	0.143	−0.328	0.319	0.443
Experience × PE -> BI	0.824	2.855	0.263	1.224	**0**.**002**
Experience × HM -> BI	−0.513	2.546	−0.804	−0.144	**0**.**005**
Experience × SI -> BI	−0.360	2.366	−0.584	−0.088	**0**.**009**

PE, performance expectancy; EF, effort expectancy; FC, facilitating conditions; SI, social influence; HT, habit; HM, hedonic motivation; BI, behavioural intention; CI, 95% Bootstrap Confidence Intervals. Bolded *p*-values represent significant interaction terms.

**Table 9:** Multi-group analysis examining moderating effect of gender.

Path relationship	Women	Men	Difference	Lower 95% (CI)	Upper 95% (CI)	*p*-value
EF -> BI	−0.045	−0.121	0.075	−0.218	0.192	0.234
FC -> BI	0.132	0.248	−0.116	−0.232	0.204	0.197
HM -> BI	0.284	0.563	−0.279	−0.344	0.315	0.083
PE -> BI	0.633	0.382	0.250	−0.284	0.367	0.120
SI -> BI	−0.026	0.007	−0.033	−0.262	0.236	0.444

PE, performance expectancy; EF, effort expectancy; FC, facilitating conditions; SI, social influence; HT, habit; HM, hedonic motivation; BI, behavioural intention; CI, 95% Bootstrap Confidence Intervals.

**Figure 3:**
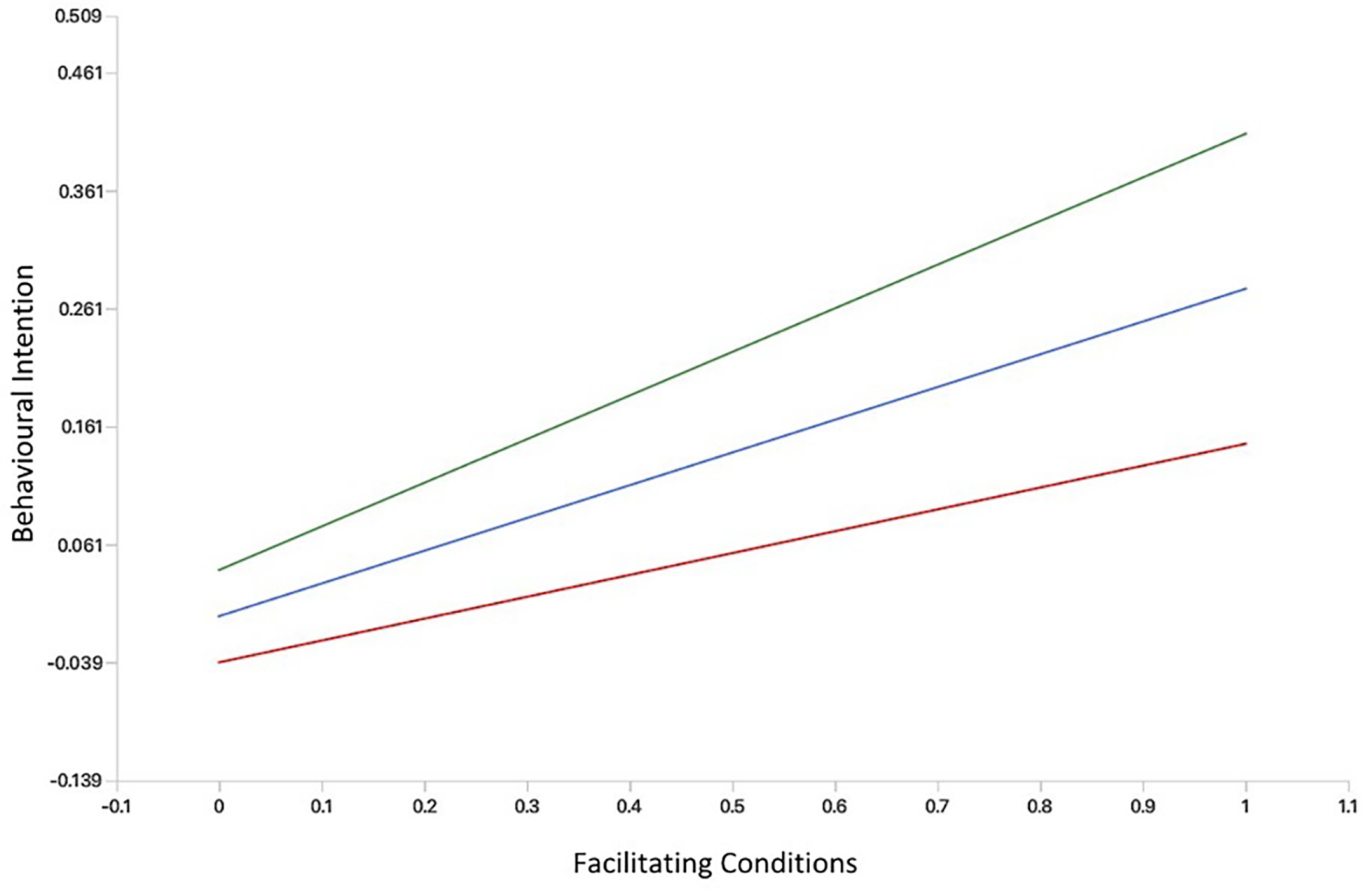
Simple slope plot showing moderating effect of Age on facilitating conditions. Green line (age = 1 *SD* above mean); Blue line (age = at mean); Red line (age = 1 *SD* below mean).

**Figure 4:**
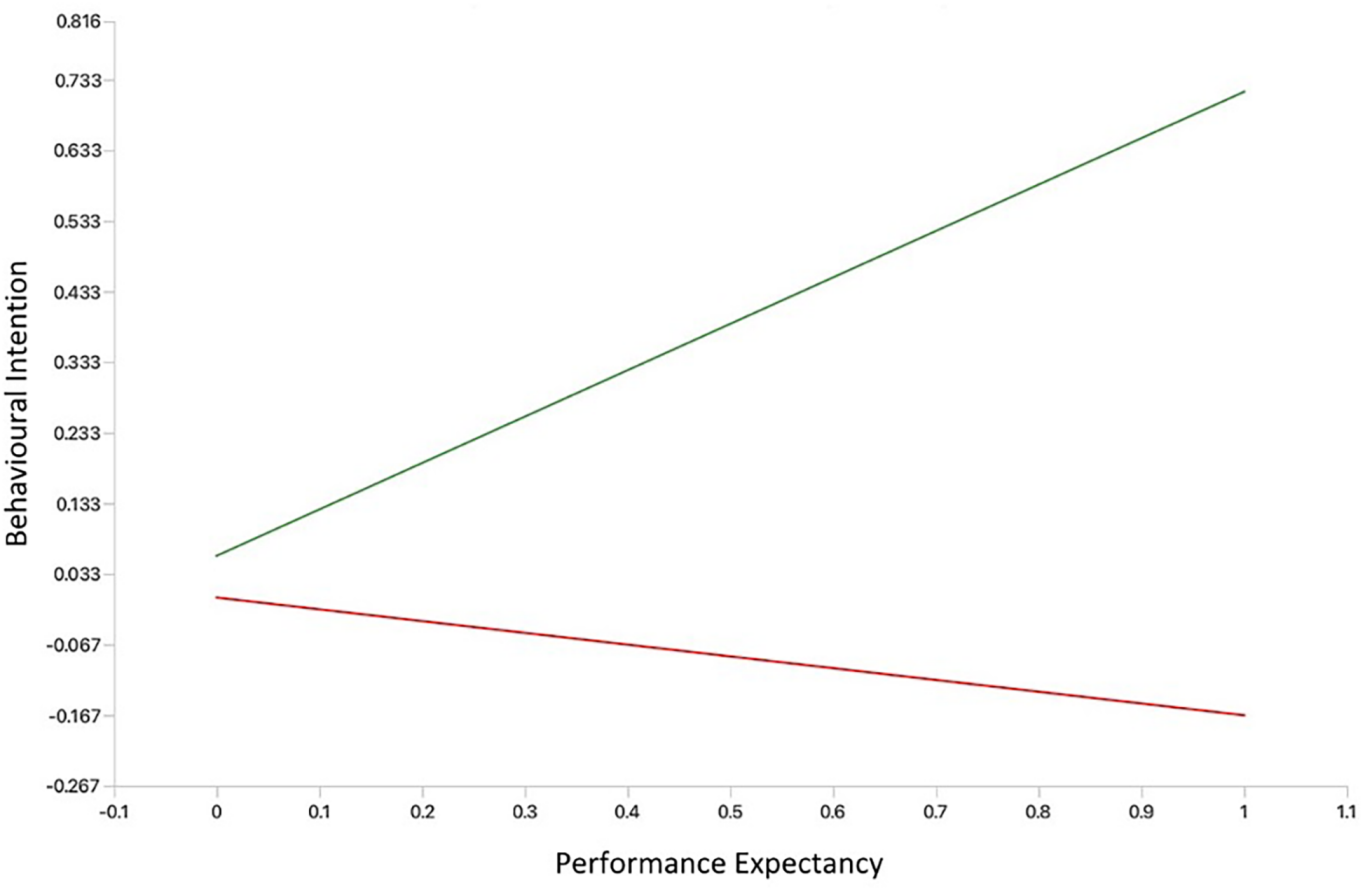
Simple slope plot showing moderating effect of experience on performance expectancy. Green line (No mental health app experience); Red line (Experience with mental health apps).

**Figure 5:**
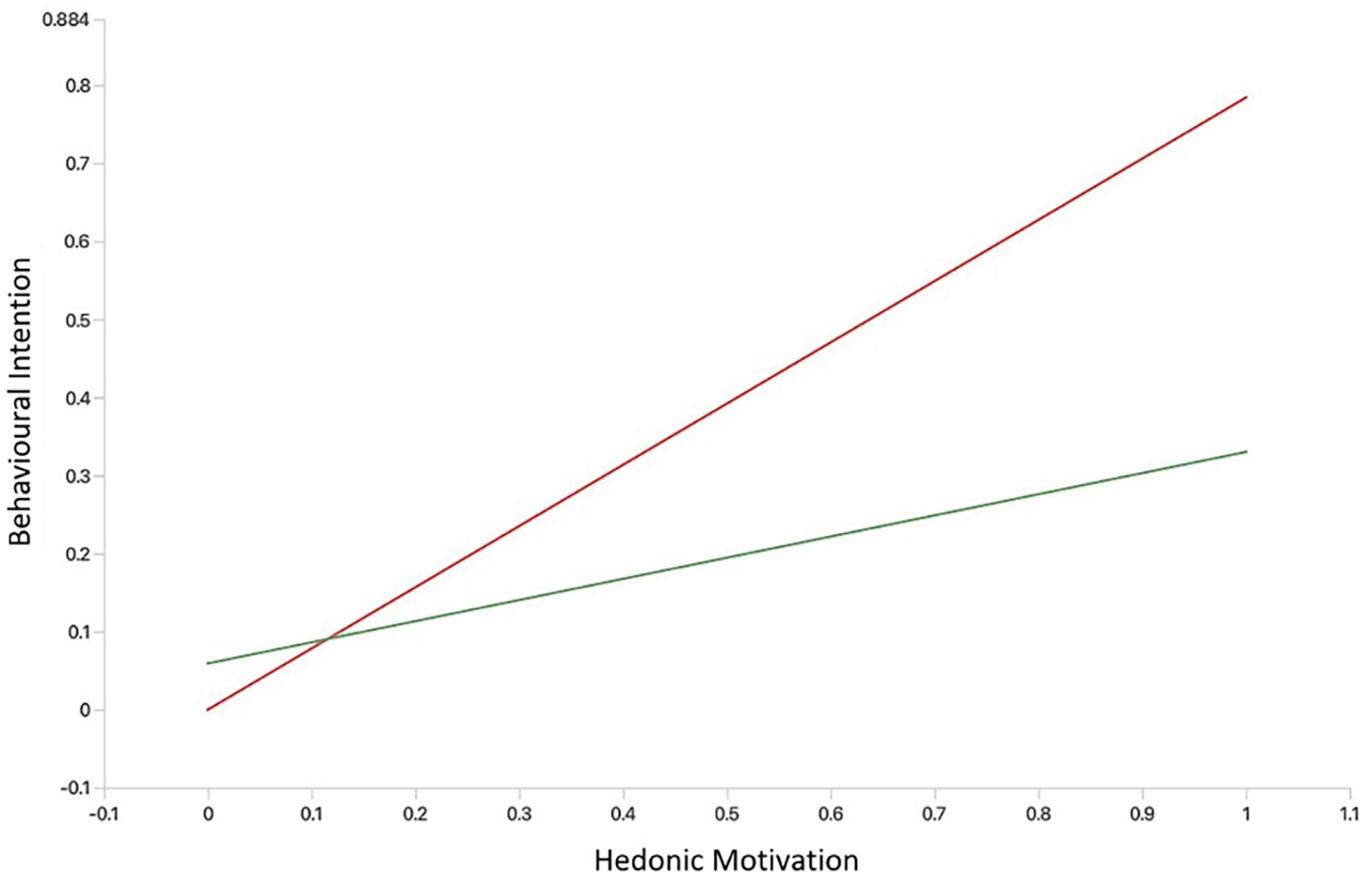
Simple slope plot showing moderating effect of experience on hedonic motivation. Green line (No mental health app experience); Red line (Experience with mental health apps).

**Figure 6:**
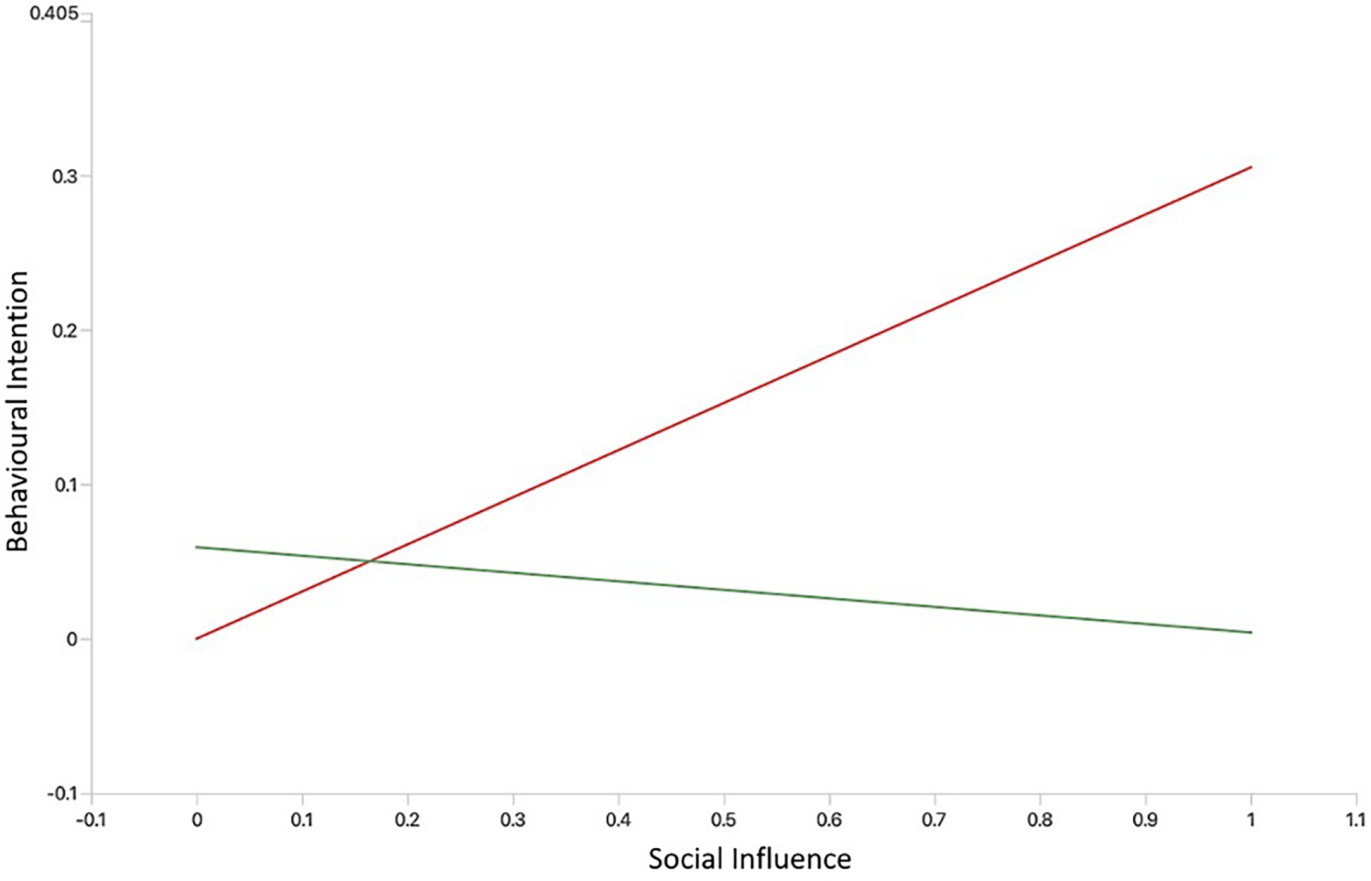
Simple slope plot showing moderating effect of experience on social influence. Green line (No mental health app experience); Red line (Experience with mental health apps).

We created simple slope plots of significant interaction effects. As age increases, the positive relationship between facilitating conditions and behavioural intention becomes stronger, and as age decreases, this relationship gets slightly weaker (see Figure 3). The relationship between performance expectancy and behavioural intention was positive and stronger for participants without MH app experience. However, among those with MH app experience, the association between performance expectancy and behavioural intention was weak and negative (see Figure 4). The positive relationship between hedonic motivation and behavioural intention is stronger for participants with MH app experience and weaker for those without prior experience (see Figure 5). The relationship between social influence and behavioural intention is strong and positive among those with MH app experience compared to those without experience, in which the relationship is weak and negative (see Figure 6).

## Discussion

Using the UTAUT2 framework, we evaluated the acceptance (behavioural intention to use) of a resilience-building MH app (JoyPop^TM^) among postsecondary students and sought to better understand factors influencing its acceptance and use. We found that most students (45.4%) accepted the JoyPop^TM^ app, which is consistent with past qualitative research on the app where users and healthcare providers reported high acceptance levels (via indicators such as usefulness and ease of use; 48, 49, 50, 52). Given that performance expectancy, facilitating conditions, and hedonic motivation significantly predicted students' intention to use the app, it is likely that students rated its acceptance highly because they had the resources necessary to use it and found it enjoyable and helpful for their MH and well-being.

### Factors influencing acceptance and Use of the JoyPop^TM^ App

We found that UTAUT2 constructs substantially influenced students' intentions to use the JoyPop^TM^ app in the future (behavioural intention). Students’ intentions to use the app and whether they have sufficient resources (e.g., devices, time; facilitating conditions) moderately impacted app use. Our model showed strong out-of-sample predictive power (i.e., utility in predicting acceptance and use of the JoyPop^TM^ app across the broader student population and similar research design contexts), supporting the external validity of our results. This pattern of results aligns with research highlighting the utility and importance of the UTAUT2 model in predicting the acceptance of mHealth apps across different contexts, populations, and countries (31, 32, 39, 73, 74).

Notably, our results also deviate from more recent, although limited, research regarding the predictive utility of the UTAUT2 model in determining users' intentions to use MH apps. For example, in past research, UTAUT2 constructs only had a minor influence on individuals' intentions to use lifestyle and therapy apps (40). Studies using the original UTAUT found that the constructs only had a small to moderate impact on whether postsecondary students intended to use popular MH apps (e.g., Headspace, Calm, Talkspace) (44). What may explain these conflicting findings is that each study was cross-sectional, and those by Schomakers et al. (40) and Holtz et al. (44) evaluated users' acceptance of groups of MH apps rather than specific apps. Further, Schomakers et al. (40) included many apps not designed explicitly for MH (e.g., diabetes-focused apps), and Holtz et al. (44) and Mitchell et al. (45) only examined the predictive utility of the original UTAUT constructs. More evaluations examining the acceptance of individual MH apps, such as the JoyPop^TM^ app, using the UTAUT2 framework are needed to substantiate its predictive utility for MH app acceptance.

Upon examining the unique influence of individual UTAUT2 constructs, we found that students' perceptions about whether the JoyPop^TM^ app could support their MH (performance expectancy) had the most substantial influence on their intentions to use the app in the future. Students' intentions were also affected by how fun and entertaining the app was perceived to be (hedonic motivation) and whether students felt they had the resources to use the app. Results are consistent with prior UTAUT2 mHealth app research among students and support the transferability and utility of the UTAUT2 in identifying and delineating the relative importance of acceptance factors across diverse student populations in different contexts and across various mHealth technologies (31, 45, 73). For instance, performance expectancy is routinely shown to be the most common and strongest predictor of mHealth app acceptance (including MH apps) among postsecondary students (31, 39, 44). mHealth and MH app acceptance among young adults and postsecondary students is also regularly shown to be influenced by hedonic motivation and facilitating conditions (31, 32, 39, 44, 45). In terms of use of the JoyPop^TM^ app, we found that it was largely dependent on students’ intentions to use it, followed by whether they felt they had sufficient resources. These results align with the large body of research demonstrating that users' intentions are the strongest determinants of mHealth app use, followed by an essential but less impactful influence of having sufficient resources (31, 39).

Although past research employing the UTAUT2 to evaluate mHealth app acceptance found that the ease of using and learning an app (effort expectancy) is a consistent predictor of acceptance (28, 32, 44), effort expectancy did not predict acceptance of the JoyPop^TM^ app. Students' skill and familiarity with smartphone apps (19, 20) and the finding that almost all participants found the JoyPop^TM^ easy to use may explain the effort expectancy result. It may be that effort expectancy only becomes a determining factor in users' intention to use the app when it becomes significantly difficult to use. This finding makes it important for JoyPop^TM^ app development teams to monitor effort expectancy levels while making adaptations to the app to ensure that the effort needed to use the app does not reach a point at which it negatively affects acceptance.

Also, we found that the opinions of important people in students' lives (social influence) did not predict acceptance of the JoyPop^TM^ app, which was inconsistent with the extensive UTAUT2 literature demonstrating that social influence often predicts mHealth app acceptance (28, 32, 45). This finding could be attributed to students experiencing less stigma because seeking support for MH is widely promoted across postsecondary institutions, most being emerging adults and basing their decisions more on their own choices and agency (1), and that users can access MH apps privately and discretely in their own time. Prior qualitative research on the JoyPop^TM^ app supports the privacy interpretation. Specifically, users perceived the app as helpful because it provided a safe and private tool to work on their MH (50). Although our moderation analysis provided important insight into the relationship between social influence and behavioural intention (discussed below), qualitative studies could be conducted to clarify why social influence does not influence students' acceptance of the JoyPop^TM^ app.

#### Moderating effects of gender, age, and experience

##### Gender

Although the literature examining the influence of gender on mHealth app acceptance is mixed (28, 31), gender has an important pooled effect on UTAUT2 relationships (31, 39). We did not find moderating effects for gender in the present study, which might be attributed to the variety of features offered through the JoyPop^TM^ app and its transdiagnostic focus on emotion regulation and resilience building. Although men and women differ on average in their coping strategies (75), the JoyPop^TM^ app offers various ways to implement adaptive coping skills. It also includes features that can be used beyond coping (e.g., Journaling to plan activities). Thus, gender may be less important because the app allows users to create individualized methods to improve their MH and build resilience. This result should be interpreted cautiously, as there were more women than men in the present sample.

##### Age

We found age moderates the relationship between facilitating conditions and behavioural intention. Older students placed a stronger emphasis on ensuring they have sufficient resources to use the app. This result was inconsistent with prior studies showing that the relationship between users' knowledge and resources to use the app and their intentions to use the app is independent of their age (31, 76). However, previous studies have primarily evaluated the acceptance of general mHealth services (e.g., websites, telehealth, wearable technologies, computer systems) targeting broad medical and health issues (e.g., metabolic and cardiovascular diseases, fitness) among adults and healthcare providers (31, 39), and no studies have examined the acceptance of an individual MH app among postsecondary students. Older students may need to become more familiar with MH apps and have more responsibilities outside of school (e.g., children), thus placing more importance on ensuring they have the resources and knowledge required to use the JoyPop^TM^ app effectively (77, 78). Older students may also be less inclined to use an app that is incompatible with the technologies they already use. Research finds that older adults are more hesitant about using novel technologies because they may be more inconvenienced by the effort required to use them, have more concerns about their utility, and may need more general awareness and knowledge about them (77, 78). Thus, older adults may need more information and support related to the utility, convenience benefits, and potential technical difficulties associated with newer technology (77, 78). This finding partly implies that to increase acceptance among older students, developers would benefit from ensuring that these students are provided with extra support and information about how to use the app (78).

##### Experience

MH app experience moderated the relationships between performance expectancy, social influence, hedonic motivation, and behavioural intention. This pattern of results is consistent with prior research showing that relationships between core UTAUT2 constructs and behavioural intention to use mHealth technologies depend on user experience (31, 35). Students with MH app experience placed less importance on how beneficial the JoyPop^TM^ app was for their MH and more on the enjoyment and entertainment it offered. One interpretation of these findings is that those with MH app experience have previously found MH apps helpful for the benefits they can provide (e.g., increased access to support and improved MH) and developed a belief that apps like JoyPop^TM^ can be valuable. Consequently, they may prioritize whether the app provides enough pleasure and fun to keep them engaged and promote continued use. Users with no MH app experience may be unsure whether the JoyPop^TM^ app can fulfill its designed purpose and place less emphasis on whether it is fun and enjoyable and prioritize whether it provides any benefit to their MH and coping skills. It is important to consider that our indicator of experience was categorical (i.e., yes or no) and that the moderating influence of experience may change depending on the specific amount of experience a user has (e.g., number of months).

Although social influence had no significant main effect on behavioural intention, we found that the impact of social influence was moderated by experience. Consistent with past research (31, 35), participants with MH app experience placed more emphasis on the opinions of significant others in their intention to use the app compared to those with no experience, in which the opinions of others had a minimal negative effect. Self-stigma (e.g., perceiving oneself as socially undesirable because of seeking MH support) (79) may help explain why MH app experience influences the relationship between social influence and behavioural intention. Compared to individuals with no experience, those with experience may have developed higher levels of self-stigma from prior MH app use and thus be more sensitive to the opinions of others. However, being influenced by significant others is not always negative because they play an essential informal role in help-seeking (80, 81). Prior users of MH apps may be positively influenced by others who know them and have seen that MH supports, like MH apps, have been helpful for them (80, 81). Although more research is needed to fully explore how others influence experienced users' acceptance of the JoyPop^TM^ app, strength-based supports, like the JoyPop^TM^ app, can reduce the potential effects of self-stigma (82).

Taken together, our results examining the moderating effects of age, gender, and experience highlight the importance of including moderators posited by the UTAUT2 to understand the acceptance of MH apps. The influence of these moderating effects across mHealth acceptance studies is mixed and depends on the context, sample, setting, and specific apps evaluated (31, 32). Interestingly, the moderating effects of age, gender, and experience may occur via three-way interactions. For example, Venkatesh et al. (35) found that the effect of effort expectancy on behavioural intention was stronger among older women (age × gender × effort expectancy). Future studies would benefit from examining higher-order interaction effects among specific MH apps.

### Limitations and suggestions for future research

The first limitation of this study is that we excluded the habit construct from the structural model because of inadequate discriminant validity. Research supports habit as a strong predictor of behavioural intention and use of mHealth apps (31, 32, 39). Our adaptations of items comprising the habit construct may have influenced discriminant validity. It will be important that future studies explore whether retaining the original items is beneficial.

A second limitation is our subjective measure of JoyPop^TM^ app use. Although studies employing the UTAUT2 to examine mHealth app acceptance often use a subjective usage measure (39), recent recommendations suggest incorporating objective usage metrics (39, 43). While we tracked the number of days participants used the JoyPop^TM^ app, we did not use this as an outcome measure and relied on a subjective measure of use because of high levels of actual usage (and lack of variation) among most of the sample. Future studies would benefit from examining acceptance over more extended periods to increase the likelihood that days used will vary enough to capture the relationship between behavioural intention and actual usage of the JoyPop^TM^ app. It would be helpful for future research to include both subjective and objective measures of app usage.

A third limitation is that we used a convenience sample comprised primarily of women. Research finds that women are more likely to use mHealth apps designed to improve emotion regulation, while men prefer accessing health-related information mHealth apps that include gamification, goal-based, and built-in tracking features (83, 84). Although we accounted for this by including gender as a moderating variable, which showed no significant differences in estimated path coefficients, unequal sample sizes across groups warrant caution when interpreting this result. Thus, it is unknown whether the results will generalize to men and the broader Canadian postsecondary population (83, 84). Future acceptance research would benefit from recruiting more balanced proportions of men and women or conducting a similar study among only men. However, the generalizability of our results to the broader student population is supported by our assessment and demonstration of the model's strong predictive power and external validity and the commonalities between the demographics in our sample and the student population in Ontario (85).

A fourth limitation concerns our one-week prospective design. Technology acceptance is a staged and temporal process, and user engagement can be complex and vary over time (86). Although having participants use the JoyPop^TM^ for one week facilitated informed perspectives regarding its acceptance, the relative importance of acceptance factors may change over extended periods of use. It will be important that future research assess acceptance at multiple time points over longer periods to better capture the process of technology acceptance and track its changing nature at varying stages of use.

### Implications

Our results support the applicability of the UTAUT2 model in evaluating and understanding the acceptance and use of MH apps. Using the UTAUT2 can facilitate MH app comparisons to determine the most important constructs influencing app engagement while providing insight into the relative impact of constructs across different MH apps. The present study can also serve as an example for future researchers examining MH app acceptance and guide the initial selection of the most appropriate and established constructs and moderators. After an app undergoes an initial evaluation using the UTAUT2, additional constructs (e.g., perceived trust) and moderators (e.g., personality traits) shown to influence acceptance and use can be added to assess whether they add predictive value.

A key implication of this study is that it can provide a valuable process to facilitate optimal app design and ensure adaptations provide the most users with as helpful and engaging an experience as possible. After examining important UTAUT2 constructs and moderators (e.g., age) influencing app acceptance and use, apps can be tailored or adapted to tap into strongly predictive constructs and nuanced user characteristics. Further, understanding which constructs do not predict acceptance and use allows developers to potentially sacrifice attributes of an app at the expense of constructs with stronger predictive strength. For example, considering our results, JoyPop^TM^ development teams could increase acceptance and use by adding a wider variety of features to improve the app's ability to enhance user resilience and adaptive coping (i.e., performance expectancy), incorporating gamification aspects and reward systems into features to increase the entertainment and enjoyability (i.e., hedonic motivation), and expanding the app to Android devices to ensure users have the resources to use the app (i.e., facilitating conditions). MH app development teams can then engage in an iterative process in which they evaluate factors influencing the acceptance of their app throughout all stages of development and implementation.

Finally, our results add to the literature suggesting that the JoyPop^TM^ app can be an engaging and helpful tool for diverse students to build resilience and improve MH and well-being. Findings support the potential integration of the app into usual campus MH services to support increasing student MH needs and reduce common barriers to care. Results also provide critical information on the JoyPop^TM^ app's acceptance, which will help inform future iterations to ensure changes address factors shown to influence its acceptance (or, at minimum, do not worsen it) to increase user engagement, long-term uptake, and the app's ability to meet the diverse needs of students.

## Conclusion

The present study was the first to employ the UTAUT2 framework to evaluate and understand factors influencing the acceptance of a resilience-building MH app (JoyPop^TM^) among postsecondary students. Most students accepted the JoyPop^TM^ app. Students' acceptance was significantly influenced by their perception of the app's utility in providing helpful and efficient MH support, how much they enjoyed and were entertained by it, and whether they had sufficient resources to use it. Findings highlight the utility and importance of employing the UTAUT2 to evaluate and understand factors influencing MH app acceptance. Results support the JoyPop^TM^ app as a potentially helpful tool to integrate into postsecondary MH services, support student well-being, and reduce common barriers to care.

## Data Availability

The raw data supporting the conclusions of this article will be made available by the authors, without undue reservation.

## Footnotes

^1^Due to insufficient variation in the number of days participants used the app (i.e., the majority of participants used the app every day; see the participants section) across the study period, we did not include days used as our primary app use outcome variable.
