# Supplementary material for: Acceptance of a mental health app (JoyPopTM) for postsecondary students: a prospective evaluation using the UTAUT2
Source: Front Digit Health. 2025 Feb 18;7:1503428. doi: 10.3389/fdgth.2025.1503428 (PMC11876143; doi:10.3389/fdgth.2025.1503428)
Supplement: Supplementary file 1 [file Supplementaryfile1.pdf]

## ***Supplementary Material***

**Supplementary File S1: Adapted Measure of the Extended Unified Theory of Acceptance and Use of Technology (UTAUT2) Constructs and Behavioural Intention (35)**

Performance Expectancy
PE1. I find the JoyPop™ app useful in my daily life.
PE2. Using the JoyPop™ app helps me accomplish things more quickly.
PE3. Using the JoyPop™ app improves my mental health and/or productivity.

Effort Expectancy
EE1. Learning how to use the JoyPop™ app is easy for me.
EE2. My interaction with the JoyPop™ app is clear and understandable.
EE3. I find the JoyPop™ app easy to use.
EE4. It is easy for me to become skillful at using the JoyPop™ app.

Social Influence
S11. People who are important to me think that I should use the JoyPop™ app.
S12. People who influence my behavior think that I should use the JoyPop™ app.
S13. People whose opinions that I value prefer that I use the JoyPop™ app.

Facilitating Conditions
FC1. I have the resources necessary to use the JoyPop™ app.
FC2. I have the knowledge necessary to use the JoyPop™ app.
FC3. The JoyPop™ app is compatible with other technologies I use.
FC4. I can get help from others when I have difficulties using the JoyPop™ app.

Hedonic Motivation
HM1. Using the JoyPop™ app is fun.
HM2. Using the JoyPop™ app is enjoyable.
HM3. Using the JoyPop™ app is very entertaining.

Habit
HT1. The use of the JoyPop™ app has become a habit for me.
HT2. I am immersed in using/accepting the JoyPop™ app.
HT3. I must use the JoyPop™ app.

Behavioral Intention
BI1. I intend to continue using the JoyPop™ app in the future.
BI2. I will always try to use the JoyPop™ app in my daily life.
BI3. I plan to continue to use the JoyPop™ app frequently.

**Supplementary Table S1: $PLS_{predict}$ Guidelines Determining Strength of Predictive Power (67, 68)**

| Predictive Power | PLS-SEM model vs. Naïve LM Benchmark Prediction Error Comparison |
|---|---|
| High | Lower RMSEs produced by PLS-SEM model on all indicators |
| Medium | Lower RMSEs produced by PLS-SEM model on majority of indicators |
| Low | Lower RMSEs produced by PLS-SEM model on minority of indicators |
| Poor | Lower RMSEs produced by PLS-SEM model on none of indicators |

*Note. PLSPredict determines predictive power by comparison Root Mean Squares Errors (RMSEs) produced by the PLS-SEM model on all dependent construct indicators to those of a naïve linear regression model (LM) benchmark.*
